# Emotional Mirror Neurons in the Rat’s Anterior Cingulate Cortex

**DOI:** 10.1016/j.cub.2019.03.024

**Published:** 2019-04-22

**Authors:** Maria Carrillo, Yinging Han, Filippo Migliorati, Ming Liu, Valeria Gazzola, Christian Keysers

**Affiliations:** 1 Social Brain Lab, Netherlands Institute for Neuroscience, Royal Netherlands Academy of Arts and Sciences, Meibergdreef 47, 1105 BA Amsterdam, the Netherlands; 2 Department of Psychology, University of Amsterdam, Nieuwe Achtergracht 166, 1018 WV Amsterdam, the Netherlands

**Keywords:** empathy, mirror neurons, emotional contagion, vicarious activity, causality, fear, pain, social behavior, rodent

## Abstract

How do the emotions of others affect us? The human anterior cingulate cortex (ACC) responds while experiencing pain in the self and witnessing pain in others, but the underlying cellular mechanisms remain poorly understood. Here we show the rat ACC (area 24) contains neurons responding when a rat experiences pain as triggered by a laser and while witnessing another rat receive footshocks. Most of these neurons do not respond to a fear-conditioned sound (CS). Deactivating this region reduces freezing while witnessing footshocks to others but not while hearing the CS. A decoder trained on spike counts while witnessing footshocks to another rat can decode stimulus intensity both while witnessing pain in another and while experiencing the pain first-hand. Mirror-like neurons thus exist in the ACC that encode the pain of others in a code shared with first-hand pain experience. A smaller population of neurons responded to witnessing footshocks to others and while hearing the CS but not while experiencing laser-triggered pain. These differential responses suggest that the ACC may contain channels that map the distress of another animal onto a mosaic of pain- and fear-sensitive channels in the observer. More experiments are necessary to determine whether painfulness and fearfulness in particular or differences in arousal or salience are responsible for these differential responses.

## Highlights

• Rat ACC contains mirror-like neurons responding to pain experience and observation
• Most do not respond to another salient negative emotion: fear
• One can decode pain intensity in the self from a pattern decoding pain in others
• Deactivating this region (area 24) impairs the social transmission of distress

## Graphical Abstract

**Figure:**
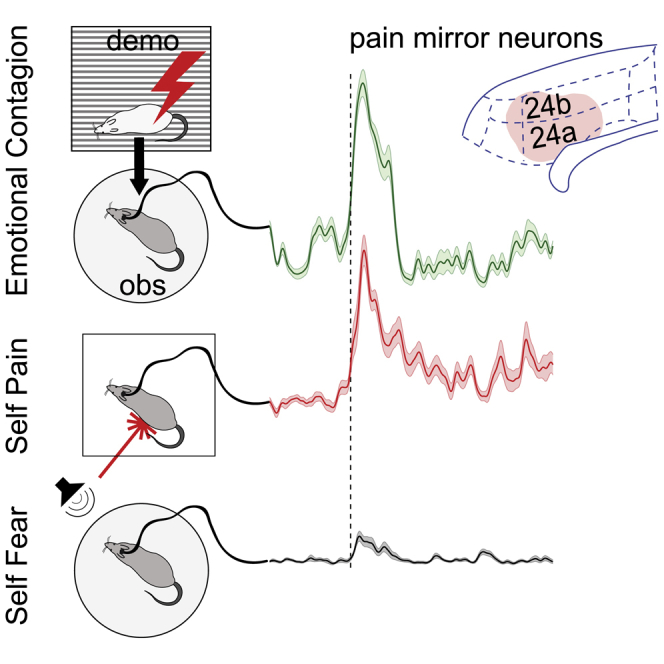

## Teaser

Carrillo et al. show the rat anterior cingulate cortex contains emotional mirror neurons that respond when a rat experiences pain and witnesses another rat in pain but not while experiencing another salient emotion, fear. After cingulate deactivation, rats show reduced distress when witnessing another receive a shock.

## Introduction

Understanding how we share the affective states of others is important for understanding social interactions [1]. Neuroimaging shows that humans recruit their anterior cingulate cortex (ACC) both while experiencing pain and, vicariously, while witnessing pain in others [2]. This vicarious activity is stronger in more empathic individuals [3] and reduced in psychopathy [4]. Reducing ACC activity using placebo or pharmacological analgesia alters empathy for pain [5, 6]. These findings make the ACC a region of particular interest in the search for a neural mechanism of affect sharing. Some suggest these neuroimaging findings reflect the existence of mirror neurons, i.e., neurons responding during the experience of pain and the perception of other people’s pain [7]. That some ACC neurons respond to the observation and experience of pain is supported by reports of one such neuron in a human patient [8] and by one report of neurons in the mouse ACC in which the immediate-early gene *arc* is more expressed following the experience of footshocks and witnessing another animal receive footshocks [9]. The functional properties of these neurons, however, remain unknown.

The selectivity of brain regions and neurons for a particular emotion is of particular interest. It has been argued that a vicarious response can only signal that someone else is in pain (as opposed to, for instance, in fear) if it has at least the following two features [10]. First, neural responses must be *selective*. If the same neuron responds to the experience of pain as much as to other salient emotions (e.g., fear), its firing cannot signal pain as different from these other emotions [10, 11]. Second, the population of neurons should employ a *common code* to signal pain in the self and in others. If the brain reads out the pain of others from the vicarious ensemble activation of a subset of its own pain neurons, then a decoder able to decode pain levels of others from ensemble activity should be able to decode pain levels in the self from the same ensemble using the same rule [12, 13]. Despite considerable efforts, fMRI experiments so far have failed to provide consistent evidence for either of these two criteria. The ACC is recruited by many salient stimuli beyond pain [10, 11]. Studies show a decoder trained to distinguish pain from no-pain trials when observed in others can decode them when experienced in the self [13] but decoders trained to distinguish different levels of pain in others fail to distinguish different levels of pain in the self [12]. That functional neuroimaging pools the activity of millions of neurons within each voxel may cause these failures.

**Figure 1:**
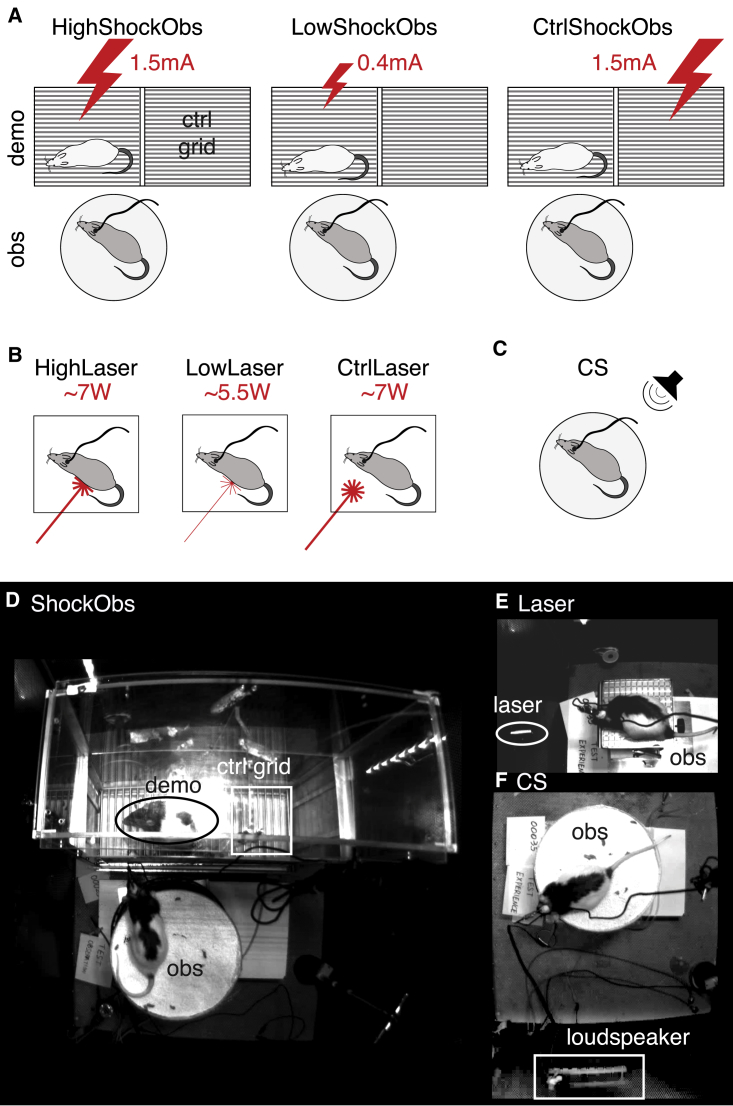
Experimental Design (A) In the ShockObs condition, the silicon probe-implanted animal (obs) sits on a circular platform (bottom) while witnessing the demonstrator (demo; top) receive high- or low-intensity shocks (big and small lightning bolts). In the control condition (CtrlShockObs), the shock is delivered to a grid next to the demonstrator and does not trigger pain. (B) In the Laser condition, the implanted animal is alone, and a CO_2_ laser (red beam) is shone on the rat’s paws or tail. Laser intensity is calibrated individually to trigger pain (HighLaser, thicker beam) or to be just below pain threshold (LowLaser, thinner beam). As a control condition, the laser is shone close to but without touching the animal (CtrlLaser). The LowShockObs and LowLaser conditions were added in the last 10/17 animals only. (C) In the CS condition, the implanted animal is alone, and a fear-conditioned pure tone is played back. (D–F) Frames from the actual video recording for ShockObs (D), Laser (E), and CS (F). See Video S1 for video excerpts of these conditions. See also Video S1.

Here, we use a previously established model of emotional contagion in which an animal observes a conspecific experience painful electroshocks [14, 15, 16, 17, 18, 19, 20] while we record multi- and single-unit activity using chronically implanted silicon probes in 17 rats. We explore whether some ACC locations and neurons are recruited during our social condition of shock observation (ShockObs; Figure 1A; Video S1). We then record activity in two separate sessions while the observer himself experiences conditions thought to trigger pain (Laser) or fear (listening to a shock-conditioned sound, CS; Figure 1B; Table 1). Following the tradition in the action-observation literature to classify mirror neurons based on their selectivity [21, 22], here we will define neurons broadly responding to the observation and experience of an emotion as emotional mirror neurons, and those that respond more narrowly to pain but not fear or fear but not pain as emotion-specific pain- or fear-mirror neurons. Here, we thus ask three questions: does the ACC contain (1) emotional mirror neurons, (2) emotion-specific mirror neurons, and (3) common coding? Thoroughly establishing specificity for an emotion would require testing neurons with a comprehensive battery of all emotions in the self and other, perfectly matched for salience and arousal. This will not be achieved in our experiment. Instead, we endeavor a step in that direction by contrasting the experience of two high-salience aversive states (pain and fear) in the self, and tentatively operationalize the terms pain- and fear-mirror neurons as those that distinguish between our pain (Laser) and fear (CS) conditions in the self.

**Table 1:** Timing of the Experiments

	Electrophysiology Experiment	Muscimol Experiment
Week 1	acclimation	acclimation
Week 2	handling	handling
Week 3	pre-exposure: shock, CS, and laser	surgery and recovery
Week 4	surgery and recovery	pre-exposure: shock and CS habituation 1; test: ShockObs
Week 5	habituation	habituation 2; test: CS
Week 6	test: ShockObs, Laser, and CS	–

Video S1. Experimental Design, Related to Figure 1 and STAR Methods

A number of specific methodological choices were made in our paradigm. We chose rats, because area 24 of the rat ACC (formally referred to as Cg1 and Cg2) is similar in cytoarchitecture and connectivity to the ACC implicated in pain empathy in humans [2, 10, 23] and is activated by the distress of others [9, 16, 24], and rats are large enough to facilitate chronic recordings in awake behaving animals. We pre-exposed the observers to footshocks 2–3 weeks before the main experiment, because having experienced electroshocks is critical in rats for showing robust signs of vicarious distress (freezing) while witnessing another animal receive electroshocks [14]. This suggests emotional contagion in this paradigm is mediated in part by sensory cues that the animal learns to decode through self-experience, with the sound and sight of the shock reactions playing significant roles [14, 16, 25]. We used footshocks to the demonstrator because this is the best characterized trigger of emotional contagion in rats. During pre-exposure, we paired the shocks with a tone to later compare responses to self-pain (Laser) and others’ pain (ShockObs) against the fear triggered by hearing this fear-conditioned tone (CS) played again. Because shocks to the implanted animal would induce artifacts in the recordings, to test responses to self-pain without compromising signal quality, instead of shocks we used a CO_2_ heat laser calibrated to trigger a nocifensive reaction, a well-characterized pain-induction method [26, 27].

In what follows, we first present the multiunit activity (MUA) from our silicon probes. MUA pools the spiking activity of thousands of neurons within ∼0.2 mm of each electrode contact [28] and is particularly stable across days [29], which is desirable given that our ShockObs, Laser, and CS conditions were recorded in sessions spread across 2 days. With this signal, we explore whether the rat ACC has locations showing activity that overlaps across observed and experienced emotions in a way that approximates the mesoscopic spatial scale of human fMRI. We then examine the activity of those single neurons that could be reliably isolated and tracked across multiple sessions to test whether overlap at the MUA level indeed reflects the presence of mirror neurons, and whether such mirror neurons are selective and instantiate a common code. Furthermore, we characterize behavioral responses during the time of MUA and single-cell responses to examine what might drive ACC responses. Finally, we will address a last question: (4) is ACC activity necessary to get contaged by the distress of another? We transiently deactivated the ACC using muscimol microinjections in a new group of animals while exposing them to HighShockObs and CS.

We find that the rat ACC indeed contains emotional mirror neurons. Most of these show a preference for one of our first-hand experiences, with the majority responding more to Laser than CS. Spike decoding provides evidence for common coding across observed and experienced pain. Deactivating this region reduces freezing while witnessing footshocks but not while hearing the CS. Together, this suggests the rat ACC maps the experience of another animal onto a mosaic of pain- and fear-sensitive neurons in the observer, and this region is necessary for emotional contagion to trigger freezing.

## Results

### Responses to the Observation and Experience of Emotions Overlap in the ACC

**Figure 2:**
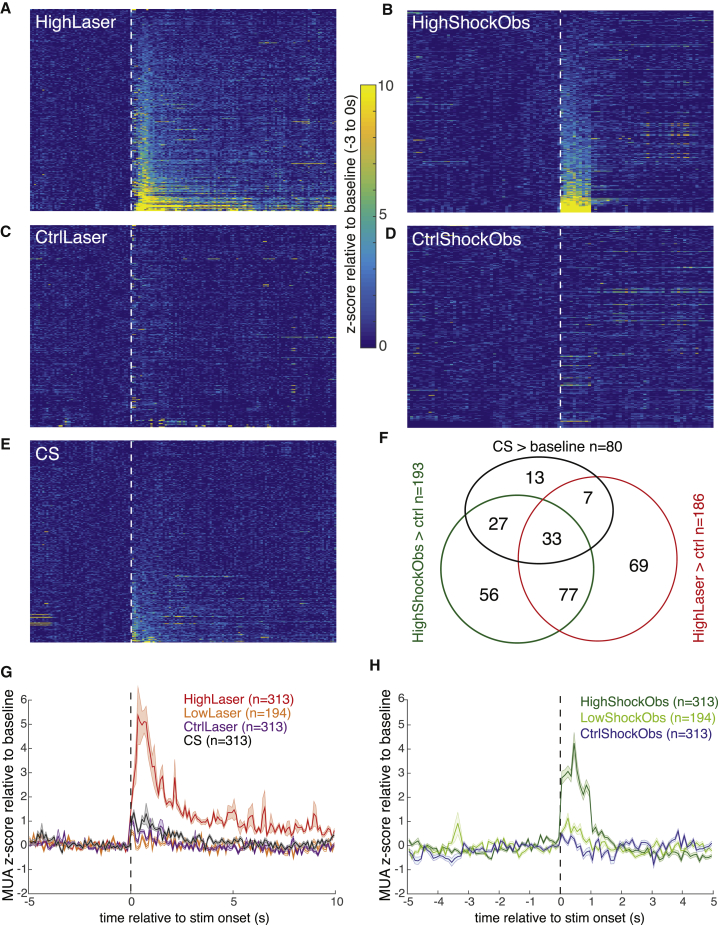
Multiunit Activity For a Figure360 author presentation of this figure, see https://doi.org/10.1016/j.cub.2019.03.024. (A–E) MUA of the 313 responsive MUA channels tested in the HighLaser (A), HighShockObs (B), CtrlLaser (C), CtrlShockObs (D), and CS (E) conditions. Each line shows the z transformed average MUA response of a channel. Z transformation was made relative to the mean and SD of the 3 s prior to each stimulus onset. Stimulus onset is shown as the dashed white line; the time axis for (A), (C), and (E) is shown in (G), and that for (B) and (D) is shown in (H). In (A) and (C), the channels are ordered in increasing average z score in the 0.3- to 1.3-s interval following stimulus onset based on the HighLaser condition, in (B) and (D) they are based on the HighShockObs, and in (E) they are based on CS. (F) Venn diagram specifying the number of MUA channels that show specific combinations of significant responses. Each cell was tested at p < 0.01 using a t test comparing MUA in HighShockObs versus CtrlShockObs (green), HighLaser versus CtrlLaser (red), and CS versus baseline (black). Numbers indicate the number of channels that show significant activations in the respective test or intersection of tests. (G) Average of (A), (C), and (E) in all 313 channels, plus the LowLaser condition from the n = 194 channels acquired in the last 10/17 animals. The shading always represents the SEM. (H) Same as in (G) for the HighShockObs, CtrlShockObs, and LowShockObs conditions. The x axis for Laser and CS is shown over a longer period to illustrate the longer MUA response. See also Figure S1. Figure360: An Author Presentation of Figure 2

At the macroscopic scale, we first explored how many channels in the ACC show MUA that overlaps across conditions. We identified responsive channels as those that show MUA increases during at least one condition. We defined the baseline period as −1.2 to −0.2 s relative to any stimulus onset, and the stimulus response window as 0 to 1 s after stimulus onset for Shock and CS conditions. For the Laser condition, we used 0.3 to 1.3 s, because the laser depends on slower-conducting fibers [30]. Because stimulus-triggered *de*activations are rare and more difficult to interpret, we focused on stimulus-triggered *activations* (i.e., stimulus responses larger than baseline), and thus used one-tailed statistics. We later also confirmed that deactivations were rare across the 425 channels we recorded over our 17 rats: only 2/425 showed deactivations following HighShockObs, 6/425 following HighLaser, and 3/425 following CS, each tested against their baseline using matched-pair, one-tailed t test at p < 0.01. In contrast, stimulus-triggered activations were observed across a majority of our channels: 313 (74%) showed increased MUA in at least one condition (matched-pair, one-tailed t test to identify stimulus-triggered activations, HighShockObs > Baseline, HighLaser > Baseline, or CS > Baseline, p < 0.01), and we then explored the time course of the MUA response to our conditions of interest (Figure 2).

With regard to our social condition, i.e., the ShockObs condition in which the other animal is the primary stimulus, many of the 313 responsive channels revealed robust responses to the HighShockObs, with a short latency and ∼1-s duration (Figures 2B, 2D, and 2H). With regard to the first-hand experiences, responses to the HighLaser, as described in the literature [30], were strong, with a slower onset and lasting for several seconds (Figures 2A, 2C, and 2G). Responses to the CS were weaker (Figures 2E and 2G), and aligned to the beginning of the CS playback (Figure S1A). This was true despite the CS triggering robust defensive responses (Figure S2). Comparing the response to the first and last 5 trials suggests some decreases in MUA magnitude with repeated presentation for CS and HighShockObs but not for HighLaser (Figure S1B). This impression is confirmed at the population level by paired t tests. Specifically, for each channel, we calculated the area under the z transformed average MUA of that channel in the experimental window, and compared this value across all 313 responsive channels in the first versus last 5 trials. This revealed a significant decrease (i.e., habituation) for HighShockObs, t_(312)_ = 5.1, p < 0.001, and CS, t_(312)_ = 3.2, p = 0.002, but not HighLaser t_(312)_ = −1.141, p = 0.25. For HighLaser, a Bayesian t test in JASP (https://jasp-stats.org) using a default one-tailed Cauchy prior provides very strong evidence for the null hypothesis of no habituation (BF_0+_ = 32).

The Venn diagram in Figure 2F reveals overlap between channels responding when emotions are observed and experienced. To ensure that responses reflect another animal’s pain (HighShockObs) or the observer’s own pain (HighLaser) and not a conditioned response to the sound of the delivery system acquired during pre-exposure, for the ShockObs and Laser conditions, we compared the response in the experimental condition against their control (Ctrl) condition. Of the 313 responsive channels, 62% (193/313) showed a socially triggered response, i.e., HighShockObs > CtrlShockObs. Much like in the human ACC, many (71%) of the 193 channels that responded in that social condition also responded when first-hand affective experiences were triggered in the rat (HighLaser > CtrlLaser or CS > baseline) and will be labeled “mirror channels” hereafter. Most of these mirror channels showed selectivity in their response to the animal’s first-hand experience: of the 110 mirror channels responding to HighLaser > CtrlLaser, the majority (77) did not respond to the CS, and of the 60 mirror channels that responded to CS > baseline, 27 did not respond to HighLaser > CtrlLaser. Only 33 of the mirror channels responded to both first-hand conditions. Laser responses were more frequent than CS responses even among the first trials, where the effect of habituation was smaller than in later trials (Figure S1C). Figure S1D finally shows that channels preferring the CS > Laser and those preferring the Laser > CS can co-exist in simultaneously recorded channels from individual animals.

In the last 10 animals, we added a lower intensity of ShockObs and Laser to our experimental design. The LowLaser intensity was chosen as a tighter control condition and involved a laser beam directed to the same body parts as in HighLaser but with an intensity reduced by 20%—an intensity at which no nocifensive behavior was apparent (Figure S2). We suspect that this laser intensity induces a feeling of warmth in the body part but we have no behavioral readout to ascertain that any sensation was evoked, and this condition thus serves as an additional control condition. The LowShockObs condition was chosen to trigger nocifensive behavior in the demonstrator, but of lesser intensity than HighShockObs to examine whether the ACC response encodes the intensity of witnessed distress in a graded fashion. Figure 2G shows the ACC responded vigorously to the Laser condition calibrated to produce nocifensive behavior, but not to the Laser condition calibrated not to produce such nocifensive behavior. The LowShockObs condition, on the other hand, did trigger noticeable but weaker responses both in the ACC (Figure 2H) and, as we will see later, in the behavior (Figures 4B and 4E).

### The ACC Contains Emotional Mirror Neurons

**Figure 3:**
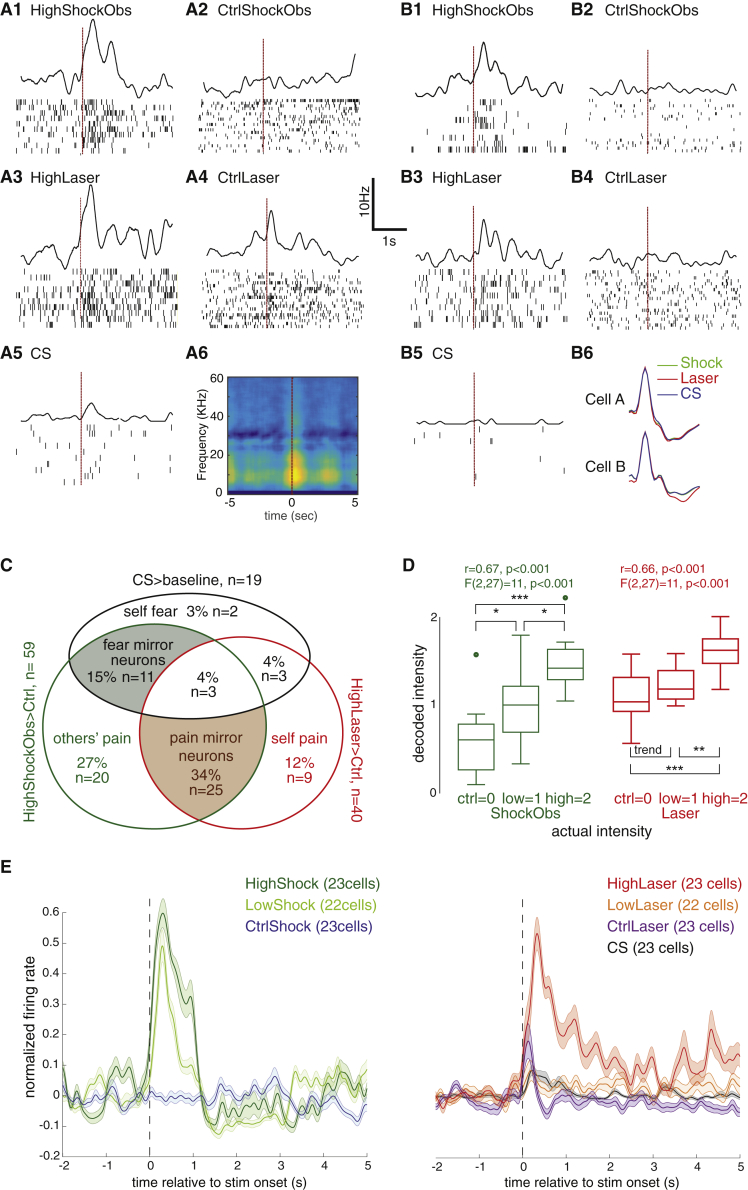
Single-Unit Activity For a Figure360 author presentation of this figure, see https://doi.org/10.1016/j.cub.2019.03.024. (A) Example cell responding to HighShockObs > Ctrl and HighLaser > Ctrl but not to CS > Baseline. (B) The same as (A) for a second example cell from a different animal. (B6) The average spike shape in each session for cells shown in (A) and (B). For cell A, we also show the spike-triggered spectrogram in the ShockObs session (A6) evidencing the broadband signature of pain squeaks. The scale bar to the left of B3 applies to all spike-density functions. (C) Venn diagram detailing the number of cells showing significant (p < 0.05) combinations of responses in HighShockObs > Ctrl, HighLaser > Ctrl, and CS > Baseline among the 73 responsive cells. (D) Whisker plot (median and quartiles) of decoded stimulus intensity based on an algorithm trained on the ShockObs session, and used to decode either leave-one-out ShockObs (green) or Laser (red) spike counts. Dots represent outliers as standard with the function Boxplot in MATLAB (MathWorks, USA). Intensities were compared using one-tailed t tests corrected for multiple comparisons using fdr; ^∗^p<0.05, ^∗∗^p<0.01, ^∗∗∗^p<0.001. (E) Mean (± SEM) spike-density function of the cells showing selective pain-mirror properties (i.e., HighShockObs > Ctrl, HighLaser > Ctrl, and HighLaser > CS). See also Figure S3. Figure360: An Author Presentation of Figure 3

To determine whether the same cells responded in different conditions, we isolated single units from the recorded signals. Spike sorting identified 84 cells spread over 13 animals that could be isolated well and followed over all three experimental sessions. In the remaining 4 animals, low electrode impedance made single-cell isolation unreliable. Using the same analysis epochs as for the MUA, among these cells, we found 73 responsive cells that showed increased spike counts in at least one condition (HighShockObs > baseline, HighLaser > baseline, or CS > baseline, non-parametric Wilcoxon test, p < 0.05). Again, there was a significant number of cells that responded in more than one condition (Figures 3A–3C).

Particularly, 59 cells (81%) were socially triggered and responded to HighShockObs > CtrlShockObs. To identify emotional mirror neurons, we explored how many of these also responded to one of the conditions in which the observer himself experienced an emotion. This was true for 28/59 (47%) that also responded to HighLaser > CtrlLaser and for 14/59 (24%) that also responded to CS > Baseline. We thus found mirror properties at the single-cell level in 66% of the ShockObs-responsive neurons.

To explore selectivity, we asked how many of these emotional mirror neurons responded differentially to Laser and CS. Only 3 of these mirror cells responded to both HighLaser > CtrlLaser and CS > baseline, whereas all others responded to only one of the first-hand experiences. For the majority of the cells (n = 25), this was to HighLaser > CtrlLaser and not to CS > baseline. Figures 3A and 3B illustrate two examples of such pain-mirror cells from different animals. In addition to a robust response to HighShockObs and HighLaser, cell A also shows a weaker transient response to CtrlLaser, a phenomenon also visible in the average MUA (Figure 2G) and which might reflect a response to the sound associated with laser delivery. To avoid this confound, we classify cells as pain responsive only if HighLaser > CtrlLaser. The selectivity of the ACC pain-mirror cells is further borne out by a direct comparison of spike counts for CS and HighLaser in the n = 25 + 3 cells that responded to HighShockObs > CtrlShockObs and HighLaser > CtrlLaser. For 23 of these 28 cells, HighLaser triggered significantly more spikes than the CS condition (Wilcoxon, p < 0.05). This provides the brain with the selectivity necessary to differentiate between states typically labeled as pain (HighLaser) and fear (CS) from the spike count of these neurons. Figure 3E illustrates the average response pattern of these 23 selective pain-mirror neurons, 22 of which were also tested with the LowShockObs and LowLaser conditions. As for the MUA, we can see a nicely graded response for ShockObs, with High > Low > Ctrl in these neurons. The response to the Laser conditions shows a transient, low-latency response to Ctrl and Low conditions that could be triggered by the sound of the delivery system, but only the HighLaser response triggered a robust, slower, and longer-lasting response expected from nociceptive fibers. A smaller proportion of mirror neurons seemed selective for the fear-inducing CS, with 11 responding significantly to CS > Baseline but not HighLaser > CtrlLaser. Only 3 indiscriminately responded to both CS and HighLaser.

A binomial distribution (59 trials at p = 0.05 each) indicates that finding 7 or more among the 59 socially responsive cells to respond to another condition is unexpected (p < 0.03), and finding 25 pain-selective mirror cells is extremely unlikely (p < 10^−14^). We therefore found significant evidence for selective emotional mirror properties in the ACC, i.e., that neurons responding to the observation of pain also respond to the experience of pain (HighLaser) but not to other, non-painful salient stimuli (CS). That so few neurons respond to all three conditions (n = 3, below what could be expected by chance) points to the fact that the ACC may contain distinct “channels” of neurons separately mapping another animal’s response to a shock onto the witness’s representations of pain (n = 25) or fear (n = 11).

Histological reconstruction of the cells showed that our recordings were mainly in area 24 extending dorsally into M2 and anteriorly into caudal area 32 (Figure S3). Exploring whether mirror cells with a particular property (pain or fear selectivity) are clustered, we tested whether their relative proportion differed across anterior-posterior coordinates or across the different cytoarchitectonic regions, but found no significant differences (Figures S3B and S3C). Mirror cells with different properties are intertwined with cells without mirror properties along the length of the explored region. If the spatial distribution of cells with these properties were similar in humans and rodents, the lack of specificity at the level of fMRI voxels [10, 11] may indeed have been the result of pooling the response of neurons with different selectivity within a voxel. To test whether selectivity is blurred at more macroscopic scales, we inspected whether the MUA (that pools activity over about 0.2 mm) shows less selectivity than the single neurons. Specifically, we used a χ^2^ test to compare the Venn diagrams obtained for MUA channels and single neurons (Figure 2F versus Figure 3C). We found a trend toward a difference (χ^2^ (6) = 12.3, p = 0.0544), with selective mirror properties, i.e., fear- or pain-mirror neurons, indeed more frequent in single neurons (37% of channels but 49% of neurons) and unselective mirror neurons, i.e., responding to ShockObs, Laser, and CS, indeed more frequent in the MUA (12% of channels but 4% of cells). However, this trend did not reach significance, and the proportion of cells and channels showing mirror properties overall (be it selective or not) was very similar in both techniques (49% in MUA and 53% in single units). Future experiments may wish to explore local field potential (LFP) activity from the same electrodes to sample signals (1) from a larger area [31] and (2) originating from events corresponding more closely to the blood-oxygen-level-dependent (BOLD) signal [32] to further constrain the interpretation of fMRI experiments on emotional contagion.

### Common Coding in the ACC

To explore the notion that the code the ACC uses to represent the distress of others is shared with that used to represent distress in the self—at least within a subpopulation of neurons—we used a decoding algorithm that can be applied to the spike count of the 69 neurons for which we have 10 trials of the High, Low, and Ctrl conditions for ShockObs and Laser. We chose to fit a generalized linear model via penalized maximum likelihood (glmnet). We first trained the algorithm to decode ShockObs spike counts, and found the resulting algorithm performs well on leave-one-out trials from the same condition (Figure 3D, green). To test common coding, the key question is: will the same algorithm decode Laser spike counts above chance without additional training? The answer is yes (Figure 3D, red), with a correlation between actual and decoded stimulus intensity of r = 0.66, t_(28)_ = 4.7, p < 0.001. This suggests that pain observation and pain experience do share a common code. An ANOVA across the 3 Laser conditions (Figure 3D, red; main effect of condition, F_(2,27)_ = 11, p < 0.001) showed that the two conditions that were calibrated not to induce nocifensive behavior (CtrlLaser and LowLaser) were decoded as of similar intensity (paired t test, p > 0.08), whereas the condition that triggered nocifensive behavior (HighLaser) was decoded as significantly more intense than either of the other two. Interestingly, training on Laser and testing on ShockObs trials did not lead to accurate cross-modal decoding: when the glmnet was trained on Laser spike counts, the leave-one-out decoding of the Laser trials worked well (r = 0.59, t_(28)_ = 3.88, p < 0.001); however, this decoder did not accurately decode the ShockObs trials (r = 0.13, t_(28)_ = 0.68, p = 0.49).

A glmnet takes the spike counts from all 69 cells, and looks for a linear combination of spike counts from as few cells as possible to decode stimulus intensity. Examination of the regression weights evidenced that the 7 neurons the glmnet selected when trained on ShockObs did contain information about Laser intensity, whereas the 4 cells the glmnet selected when trained on Laser did not contain information about ShockObs intensity. This suggests an asymmetry in ACC representations, with those neurons providing the clearest ShockObs signals also carrying Laser intensity signals, but those providing the clearest Laser signals not necessarily also encoding ShockObs. To double check that within the mirror neurons, common coding operates in both directions, we replicated the analysis in both directions when feeding the glmnet only the 11 neurons for which we had 10 trials for the High, Low, and Ctrl conditions of ShockObs and Laser, and for which we had a significant response in both conditions (High > Ctrl). When training on ShockObs, Leave-one-out decoding of ShockObs worked as well as when considering all 69 cells (r = 0.6, t_(28)_ = 4, p < 0.001), and decoding of Laser trials also worked as well as with all 69 cells (r = 0.67, t_(28)_ = 4.9, p < 0.001). Training on Laser led to good decoding of leave-one-out laser trials (r = 0.52, t_(28)_ = 3.2, p < 0.005) and to above-chance ShockObs decoding (r = 0.6, t_(28)_ = 4, p < 0.001). This confirms that there is a population of neurons in the ACC that codes ShockObs and Laser in the same code but, considering the results from the 69 neurons, this common coding is not implemented in all neurons providing strong Laser intensity signals.

### The Demonstrators Squeak and Jump while the ACC Responds Maximally

**Figure 4:**
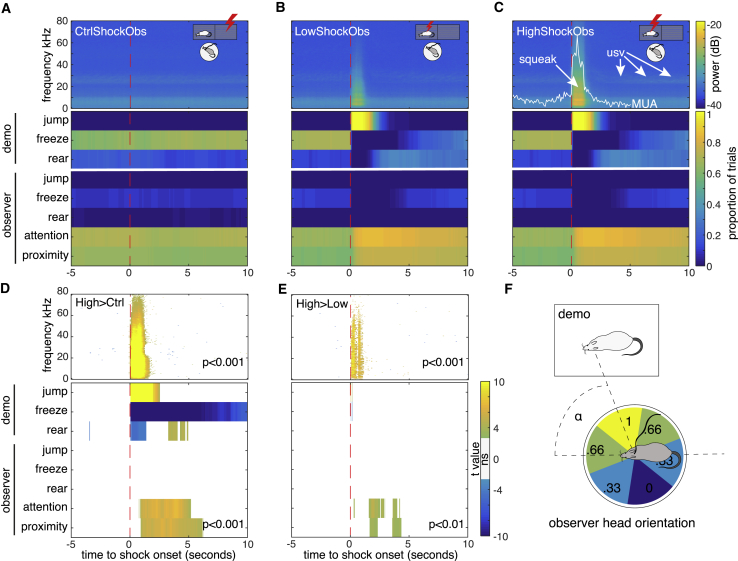
Behavioral Scoring of the Shock Conditions For a Figure360 author presentation of this figure, see https://doi.org/10.1016/j.cub.2019.03.024. (A–C) Grand averaged shock-triggered audio spectrogram (top) and ethogram (bottom) obtained by averaging all trials and all animals for CtrlShockObs (A), LowShockObs (B) and HighShockObs (C). Note the broadband signal occurring in the 1 s post-stimulus. This includes the pain squeak and the rattling of the cage triggered by the jump. Peak sound-pressure levels during the squeaks in the HighShock condition were ∼87 dB. (C) We also show the HighShockObs MUA response as in Figure 2H for comparison. (D) Random-effect comparison High > CtrlShockObs done by averaging all the trials per animal, and then using a matched-pair t test (n = 17 animals) pixel per pixel. (E) Same for High > LowShockObs for the 10 animals for which LowShockObs was tested. Tests are thresholded at p < 0.001, except for the n = 10 animal ethogram comparison in which no difference survives at p < 0.001. (F) Attention was quantified based on the angle α between observer head orientation and demonstrator with ± 30° considered maximal (=1) and 180 ± 30° considered minimal (=0). In the illustrated example, α = 70°, and the attention would be scored as 0.66. See also Figure S2. Figure360: An Author Presentation of Figure 4

What stimulus may have triggered the ACC response in the observers during the HighShock condition? Given that the shock was not delivered to the implanted animal, the stimulus must have originated from the demonstrator. Examining the behavior of the demonstrator during the 0- to 1-s interval of maximal ACC response revealed that jumping and squeaking were salient behaviors that were timed much as the ACC MUA response itself (Figure 4; Video S1). This is visible in the spectrogram of the sound recording as a broadband signal, and in the behavior as a dramatic increase in jumping. The frequency of jumping and intensity of squeaking scaled with shock amplitude (HighShockObs > LowShockObs > CtrlShockObs; Figures 4D and 4E) much like the MUA (Figure 2H) and spiking (Figure 3E) and temporarily interrupted the other behaviors (freezing and rearing). Although ultrasonic vocalizations around 22 kHz were also apparent following the administration of shocks, they were not specific to the 0- to 1-s window and thus cannot explain the timing of the ACC response. This makes the jumping and/or squeaking of the demonstrators seem the most likely trigger of the ACC response to HighShockObs. The observers’ actions in response to witnessing the shock included turning and walking toward the demonstrator (i.e., attention and proximity in Figures 4D and 4E).

HighLaser triggered the well-described nocifensive reactions to a laser, including rapid paw retraction and licking and rapid turning around (Figures S2B and S2C; Video S1) [31], but did not trigger squeaking similar to that in HighShockObs (Figure S2A). The response of pain-mirror neurons to HighShockObs and HighLaser (Figures 3A, 3B, and 3E) thus cannot be explained by hearing squeaking in both conditions, and must reflect a less trivial association of two physically different stimuli: one signaling the pain of another via exteroception and one signaling potential damage to the animal’s own body via nociceptive afferents. Behavioral responses to LowLaser were similar to those to CtrlLaser, and only included orienting, which could be explained by hearing the clicking of the button that delivered the laser (Figures S2B and S2C). Responses to the CS were characterized by freezing replacing the lying down and grooming that characterized baseline activity (Figure S2E). Freezing was not restricted to the actual playback of the CS but persisted during the interval between stimuli. Unlike the MUA response that decreased in the last compared to the first trials (Figure S1), freezing increased slightly with time (Figure S2E).

To further explore what stimulus may have triggered the neural response in the HighShockObs condition, we also computed spike-triggered average spectrograms, which revealed the broadband signal typical of pain squeaks to co-occur with moments of high spiking (Figure 3A6).

### The ACC Is Necessary for ShockObs- but Not CS-Triggered Freezing

**Figure 5:**
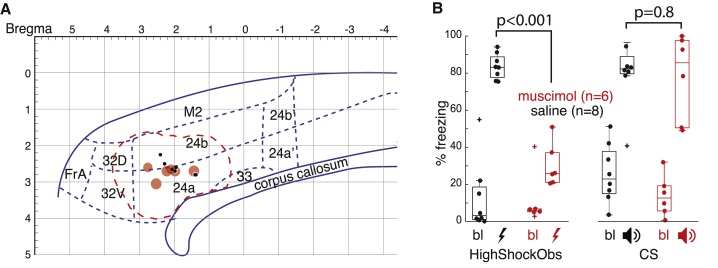
Behavioral Consequences of ACC Muscimol Deactivation (A) Locations of the n = 6 muscimol (red) and n = 8 saline (black) injections on a sagittal view of the rat cingulate, based on the anatomical divisions in [33]. The red dashed line represents the likely spread of the muscimol based on a 1-mm radius [34]. (B) Whisker plot (median and quartiles) of the freezing levels during baseline (bl) or experimental periods (ShockObs or CS playback iconized as lightning bolts and loudspeakers) in the two groups of animals. p values refer to uncorrected, two-sample two-tailed t tests across the two groups. Note that the data from the ShockObs but not the CS condition are also used to explore how this affects the behavior of the demonstrator in [35].

Finally, to test whether the ACC is necessary to trigger vicarious nocifensive behavior in the rat, we bilaterally injected muscimol or saline, into area 24 of two new small groups of observers (Figure 5A) and quantified their socially triggered freezing in response to a HighShockObs condition and to a CS playback in separate sessions. Histological reconstructions confirmed that our canulae were in area 24 and, considering an approximate radius of muscimol effect of 1 mm for the volume we injected (based on [34]; see red outline in Figure 5A), our deactivations overlap with where we found pain-mirror neurons (Figure S3). In line with previous observations in mice [16], we found that although both groups showed increases in freezing in both HighShockObs and CS sessions relative to their baselines (Figure 5B; all one-tailed, paired t test, t > 5.6, all p < 0.001, with degrees of freedom [df] 5 and 7 for muscimol and saline, respectively), and freezing was similar in the saline group for HighShockObs and CS (t_(7)_ = 0.6, p = 0.55), the socially triggered freezing (Shock) was reduced in the muscimol compared to the saline group (t_(12)_ = 10.7, p < 0.001). This was not true in the non-social condition (CS; t_(12)_ = 0.17, p > 0.8). The necessity of the ACC for socially rather than non-socially triggered freezing was confirmed by a mixed ANOVA with 2 groups (Saline versus Muscimol) × 2 sessions (Shock versus CS) × 2 Epochs (baseline versus Shock or CS) that yielded a significant 3 way interaction (F_(1,12)_ = 17, p < 0.001).

## Discussion

Our data show the rat ACC contains mirror-like multiunit and single unit activity with spiking increases during shock observation and first-hand experiences (Laser or CS). A decoding scheme trained to decode the intensity of another rat’s experience can decode the intensity of the rat’s own pain experience. Importantly, for the majority of multiunit channels and neurons, there was evidence for selectivity for the experience of laser-triggered pain over that of CS-triggered fear. Deactivating this region reduces socially triggered freezing without compromising freezing to non-social danger signals (CS).

Although it is difficult to attribute human emotional labels to rodents [32], CS is the prototypical procedure to trigger *fear*, whereas CO_2_ heat lasers are a gold-standard method for inducing *pain* [26, 27]. That many MUA channels and neurons responding to shock observation respond to the laser but not the CS suggests that shock observation may be predominantly mapped onto a representation of *pain* in the self. This dovetails with the fact that the behavioral signature most associated with the response, the squeak, is considered a highly specific pain signal [36]. The vicarious activation of ACC nociceptive neurons may then prime nocifensive behaviors in the observer, preparing it to cope with the same source of harm, including orienting toward the danger (Figure 4) and elevated freezing (Figure 5B) often reported in such paradigms [14, 15]. That an, albeit probably smaller, proportion of shock-responsive neurons preferentially respond to the CS suggests that the observer’s ACC may actually map the Shock observation onto a hybrid neural ensemble composed of a majority of pain and a minority of fear representations. That ACC deactivation compromised freezing to shock observation but not CS parallels the higher recruitment of the ACC to shock observation compared to CS.

An important question is to explore whether the ACC responses to shock observation are a cause for the observer’s emotional reaction to the distress of the other or rather a downstream representation of the observer’s reaction. That deactivation of the ACC impairs vicarious freezing suggests that it plays a causal role. Future experiments that selectively modulate activity in mirror neurons within the ACC rather than the ACC more generally will be key to addressing this question.

Our study has limitations that qualify our conclusions and invite future experiments. First, establishing that a neuron is selective for pain requires excluding that it responds to any other non-painful but equally salient emotion [10]. Showing that a number of our neurons respond to ShockObs and Laser but not, or less, to CS is but a first step in that direction. Future experiments in which a richer set of physiological parameters are collected (e.g., startle potentiation, heart-rate variability, pupil diameter) while animals are submitted to a wider range of stimuli, including non-painful conditions as salient as the painful conditions, are needed to gain a finer-grained understanding of the dimensions encoded in ACC mirror neurons [10]. That the CS and ShockObs conditions triggered similar levels of freezing in the muscimol experiment suggests they are matched along at least one indicator of negative affective relevance. Another relevant dimension meriting further investigation is the imminence of a stimulus: HighLaser represents an immediate nociceptive stimulus, whereas CS announces the likely arrival of a painful event in the future. This difference in imminence is perhaps intrinsic to the difference between pain and fear, but varying this dimension systematically could shed further light on the selectivity of ACC neurons. Contrasting our findings in area 24 (and, to a lesser extent, M2 and caudal area 32) with recordings and lesions in area 25 and more anterior parts of area 32 (also known as infralimbic and prelimbic [23] and to be involved in fear conditioning to tones [37, 38]) would sharpen our understanding of how selectivity for pain and fear coexist in the medial prefrontal cortex.

Second, the CS condition was collected a day after the ShockObs and Laser conditions for the animals to recover from the previous negative effect. Single cells could thus have drifted away from the electrodes overnight, creating a bias against the CS condition. This should apply less to the MUA data, known to be stable over time [28, 29], and that muscimol impaired freezing to ShockObs but not CS converges to suggest that losing cells over time is unlikely to entirely explain the scarcity of CS effects across all our measures.

Third, our animals showed unusually low freezing during the electrophysiological ShockObs condition compared to previous behavioral experiments and the present muscimol study [14, 15, 35]. Traditionally, we tested animals in the week following pre-exposure, in a two-compartment cage resembling that during pre-exposure and without tethering [14, 15, 35]. For electrophysiology, we introduced 2 additional weeks of habituation, placed the observer on a plastic cylinder to avoid electrical noise, and tethered the animal. This made the electrophysiological context more distinct from the initial pre-exposure and thereby reduced contextual danger cues. Such changes in context are known to reduce freezing in fear conditioning [39], and we believe this to have reduced the propensity to freeze. That the ACC nevertheless encoded ShockObs vigorously is notable, but experiments that quantify ACC responses as a function of the remoteness (in time and contextual similarity) of pre-exposure will shed light onto what the ACC represents: if ACC responses decrease with increasing safety cues, they are more likely to represent the observer’s personal risk assessment [35]. If responses remain constant, they are more likely to represent the distress of the other. Varying the similarity between the noxious stimuli used during pre-exposure and testing would illuminate a similar question from a different angle: what exactly does the observer learn during pre-exposure? Would pre-exposure with Laser suffice to make the observer sensitive to seeing another animal experience footshocks? Must there be a tighter match between the bodily reactions produced during pre-exposure and observation? In a Hebbian learning model, we predict that to hear himself jump and squeak while in pain during pre-exposure is what allows pain representations in the cingulate to bind with sensory synaptic input representing the sound of squeaking and cage rattling [14, 40, 41]. These connections later recruit ACC neurons while hearing the demonstrator produce these sounds, a notion similar to auto-conditioning in the seminal work of Church [42]. If this prediction is true, Laser, which did not trigger squeaking, would not be as effective a pre-exposure stimulus for later ShockObs.

Finally, there is an important distinction between emotional contagion and empathy. Recruiting neurons involved in one’s own experience of pain while witnessing the pain of others could suffice to trigger emotional contagion—feeling the distress that the observed animal feels—and can prepare the observer to face the danger that afflicted the demonstrator [35]. This, however, does not provide evidence that the observer understands that this vicarious pain is experienced by a specific other animal—as empathy proper would require. This distinction is particularly relevant in relation to observations of pro-social behavior and targeted helping in rodents [1, 43, 44], and invites future experiments that explore whether deactivating ACC mirror neurons influence the willingness of a rat to help another.

In summary, our study shows that the principle of selective mirroring discovered in the motor system while monkeys view or listen to the emotionally neutral actions of others [21, 45] also applies to how mammals process the affective signals of others, and paves the way to a mechanistic exploration of emotional contagion. It is notable that the brain region in which we find this mechanism (region 24 in [23]) is similar in location, cytoarchitecture, and connectivity to the location of the human cingulate in which fMRI studies have revealed an increase in BOLD signal during both pain observation and experience [2, 23]. If one embraces the notion that mammals may share a common neural mechanism for emotional contagion [1, 46], this finding is relevant to the neural basis of human intersubjectivity [7, 10, 47].

## STAR★Methods

### Key Resources Table

**Table:** 

REAGENT or RESOURCE	SOURCE	IDENTIFIER
**Chemicals, Peptides, and Recombinant Proteins**		

Muscimol	Sigma-Aldrich	Cat#M1523

**Deposited Data**		

Data and Inhouse Software	This Paper	Mendeley Data, https://doi.org/10.17632/rkt957v4n4.1

**Experimental Models: Organisms/Strains**		

Long Evan rats	Janvier, France	RjOrl:LE

**Software and Algorithms**		

MATLAB	https://www.mathworks.com/products/matlab/whatsnew.html	R2015a
Behavioral observation research Interactive software	Open source	http://www.boris.unito.it/
Fieldtrip toolbox	Open source	http://www.fieldtriptoolbox.org/
Avisoft	https://www.avisoft.com/	10101
IBM SPSS statistics	https://www.ibm.com/nl-en/products/spss-modeler/	25
GLMnet	https://cran.r-project.org/web/packages/glmnet/index.html	Version 2.0-16

### Contact for Reagent and Resource Sharing

Further information and requests for resources and reagents should be directed to and be fulfilled by the Lead Contact, Christian Keysers (c.keysers@nin.knaw.nl).

### Experimental Model and Subject Details

34 (for the electrophysiology experiment) and 60 (for the Muscimol experiment) healthy and immunocompetent male Long Evan rats (6-8weeks old/250-350 g) were obtained from Janvier, France. Animals were randomly assigned to different roles, consisting of 17 observers and 17 shock demonstrators for the electrophysiology experiment and saline control group (n = 30, 15 observers and 15 demonstrators) or muscimol group (n = 30,15 observers and 15 demonstrators) for the muscimol experiment. For the muscimol experiment, 8 saline and 6 muscimol observers could be included in the final analysis after removing those deceased during or after surgery, those with damaged or clogged canulae and those in which histological reconstruction revealed damage to the corpus callosum (see below). Upon arrival all animals from the electrophysiology experiment were socially housed in type IV cages and all animals from the muscimol experiment were housed in observer-demonstrator dyads in type III cages. Animals from both experiments were housed with corn cob bedding at ambient room temperature (22-24°C, 55% relative humidity, SPF), on a reversed 12:12 light:dark cycle (lights off at 07:00). Food and water were provided *ad libitum*. To protect the electrophysiology implant, following surgical implantation of the silicone probes in the observers, animals were placed in modified housing, which consisted of two compartments separated by stainless steel bars. All experimental procedures were pre-approved by the Centrale Commissie Dierproeven of the Netherlands (AVD801002015105) and by the welfare body of the Netherlands institute for Neuroscience (IVD, protocol number of electrophysiology experiment NIN161107; protocol number of muscimol experiment NIN151104).

### Method Details

#### Test setups

For the electrophysiology experiment, all habituations and tests were conducted inside a faraday cage, in dim red light during the dark part of the circadian rhythm, with background radio turned on. Three different set-ups were used based on condition (Figure 1; Video S1). During the shock observation test (ShockObs. Figure 1A), the observers were placed on an elevated circular platform (H:70cm, 30 cm diameter), surrounded by a transparent plastic wall of 2 cm in height, and with bedding from the observer’s home cage. The demonstrator’s testing box consisted of two chambers separated by a perforated Plexiglas divider (each: L24cm xW:25cm x H:34cm) with stainless steel grid floors. The cage was positioned close to the observer platform with the chamber containing the demonstrator closest to the observer to ensure it was clearly visible to the observer. The wall facing the observer’s platform was made of fine wire mesh (Med associates Inc, USA). During the fear conditioning recall test (CS, Figure 1C), the cage of the demonstrator was removed, and the observer placed on the same elevated platform as for shock observation, with a buzzer-like loudspeaker playing the conditioned cue (CS) placed ∼30cm away from the platform. Lastly, the observer experience of the heat laser (Laser, Figure 1B) was conducted on a rectangular stainless-steel metallic platform (15cmx15cm), elevated 30cm and with a 0.5cm fence. The CO_2_ laser was placed outside the faraday cage and the arm used for delivering the heat pulses protruded into the faraday cage, with its tip 15cm away from the observer’s platform. During all tests, behavior, vocalizations and neural activity were recorded using a top and side video camera (Basler acA1300 and mediarecorder software, Noldus, Netherlands), a condenser ultrasound microphone (Avisoft-bioacustics, CM16/CMPA, Germany) and an electrophysiology acquisition system (digital lynx SX and cheetah software, Neuralynx, USA), respectively. To avoid contextual fear, the test pre-exposure was done in a two compartment cage that was different from that used in any of the electrophysiological testing: a two chamber box with angled soft plastic walls (each: L31cm xW:24cm (bottom) L40xW:31(top) x H:44cm, Video S1) separated by a perforated Plexiglas divider with stainless steal grid floors. This pre-exposure box was also washed with a differently scented soap, the background radio was turned off, and light intensity was higher to prevent generalization across sessions.

For the muscimol experiment, the pre-exposure was conducted in the same apparatus as described for the electrophysiology experiment. The testing box consisted of two chambers (each: L24cm xW:25cm x H:34cm) divided by a perforated Plexiglas divider, with a stainless-steel grid as a floor on the side of the demonstrator and a plastic platform on the side of the observer (Med associates Inc, USA).

#### Experimental procedures

##### Acclimation and pre-exposure

Upon arrival, all animals were allowed to acclimate to the colony room for 7 days (week 1, Table 1). To reduce stress and habituate animals to the researchers, during the second week (week 2) all animals were handled every other day for 3 min per day. For the electrophysiology experiment, during week 3, to prepare and familiarize the observers with the conditions they would encounter during the test days (ShockObs, CS, and Laser), the observers experienced three types of stimuli: footshocks, fear conditioning and CO_2_ heat laser. The footshocks and fear conditioning were combined into a single pre-exposure session. The observer animal was put into one compartment of the above-described pre-exposure box and a 10 min baseline was followed by the presentation of five 20 s tones (3KHz, 70dB), each associated with the delivery of a 1 s shock (0.8mA) during the last second of the tone presentation (1 s at 0.8mA with 60 s inter-shock interval; shocker model ENV-414 from Med associates, Inc). To prepare the observer for the laser condition it was important to first measure the pain threshold for each animal, which was determined as the stimulation level at which the animal showed consistent paw retraction and/or licking. A laser pre-exposure session was then performed, in which, after a 2 min baseline, a CO_2_ heat laser (CL15 model:M3) was used to deliver 5 pulses (wavelength 10.6μm, 200ms, at 60%–70% of the total laser power of 15W, beam diameter < 2mm) aimed at the paws or tail, with a random inter-stimulus interval of 24 to 36 s. Shock demonstrators were left in their home cage ensuring they will be naive to the stimuli on test day. For the muscimol experiment, the shock pre-exposure was conducted using the same protocol as that of the electrophysiology but no conditioning tone was presented.

##### Habituations and Surgery

For the electrophysiology experiment, on week 4, observers underwent a surgical procedure for the unilateral implantation of a multi-shank silicon probe (Atlas Neuroengineering, Belgium, E32-400-SSL4-500), targeting the right anterior cingulate cortex (ACC). Buprenorphine was used for pain relief (30 mins prior to surgery, s.c. 0.01-0.05mg/kg) and isoflurane as anesthetic (4% for induction and 0.8%–2% for maintenance). Body temperature and other physiological parameters were monitored throughout the surgery. Once animals were deeply anesthetized, they were placed in a stereotaxic apparatus, six screws were attached to the skull (two of them used to connect the ground wires), a craniotomy was performed (≈4-4.5mm in diameter) and the probe was lowered to the target area (centered at Bregma AP:0.96mm, ML:0.3mm, DV:-3mm, angle from vertical: 20°) and secured using multipurpose cement (GC Fuji PLUS capsules, GC Europe N.V., Belgium). After the surgery, animals received Atipamezole (0.5mg, ip) and Metacam (1mg/kg) and were placed in an incubator until they woke up. Thereafter, they were placed in a modified homecage and received wet food until behavior and weight recovered. Behavior, state of the incision and weight was monitored daily for 3 days and once a week thereafter. Animals were allowed to recuperate for at least 7 days prior to test start. For the rest of week 4 and 5, observers and demonstrators were habituated to the experimental setups for five days (20 min/day/setup). On the last three habituation sessions, observers were tethered to the electrophysiology recording system. For the muscimol experiment, cannulae were implanted into the ACC, targeting area 24. All animals were anesthetized using isoflurane. The animals were then positioned in a stereotaxic frame with blunt-tipped ear bars, and a midline incision was made. Six holes were drilled (2 for anchoring screws and 1 for the cannula per hemisphere). Two single guide-cannulas (62001; RWD Life Science Co., Ltd) were implanted targeting bilateral ACC (AP, +1.7; ML, ± 1.6; DV, +1.8 mm with a 20° angle from the surface of the skull, Paxinos and Watson, 1998) and chronically attached in the observer animals with a thin layer of acrylic cement (Super-Bond C & B, Sun Medical Co. Ltd., Shiga, Japan) and thick layers of acrylic cement (Simplex Rapid, Kemdent, UK). To prevent clogging of the guide cannula, a dummy cannula (62101; RWD Life Science Co., Ltd) was inserted and secured until the microinjection was administered. After a week of recovery, observers were habituated to fake micro-infusions and to the experimental setup for the ShockObs condition with their demonstrator for 20 min.

##### Testing of electrophysiology experiment

On week 6, the electrophysiological recording and test sessions were conducted during two consecutive days. Day one included ShockObs followed by Laser and on day two, the CS recall test. Separation of the tests onto two different days was to ensure that baseline activity in the ACC during the second aversive experience does not reflect a carry-over from the previous first-hand experience. Laser preceded the CS session because the question of whether the same cells respond to pain observation and experience was primary, and the question of selectivity with regard to CS secondary. That fewer cells respond to CS than Laser should therefore be interpreted cautiously. *ShockObs:* test started with a 12-minute baseline, followed by the observer witnessing the demonstrator experience different shock intensities. In the first 7 animals we tested two conditions: 10 high intensity (HighShockObs, 1.5mA) 1 s shocks and 10 control 1 s shocks (CtrlShockObs) of same intensity delivered to a grid in the compartment adjacent to that of the demonstrator (intershock interval 60 or 90 s). In the last 10 animals we added 10 additional 1 s shocks of low intensity (LowShockObs, 0.4mA) and increased the number of control shocks to 20. 0.4mA was chosen because this still leads to a visible and audible reaction of the demonstrator but one that is clearly less intense than that at 1.5mA (Figure 4). *Laser:* test was conducted at least 20 mins after shock observation and it consisted of a 5 min baseline followed by the laser stimulation trials. As for Shock, in the first 7 animals, we tested 2 conditions consisting of 10 high intensity stimulations (HighLaser, 50%–70% of the laser power depending on the pre-determined pain threshold per animal), and 10 control stimulations targeted near but not on the animal (CtrlLaser). Each laser stimulation lasted 200ms, and were separated by an inter-laser interval of either 24 or 36 s. In the last 10 animals we added an intermediate intensity for a total of 40 trials: 10 high intensity stimulations, 10 low intensity stimulations (LowLaser, 20% less than the HighLaser intensity), and 20 control stimulations. In the high and low intensity trials, the paw or tail of the animal were targeted. Using 20% less laser intensity was chosen to have a more restrictive control stimulus in which the laser is shone onto the animal (probably inducing a termal sensation) without inducing pain. The lack of clear nocifensive behavior (no paw licking or retraction) supports the absence of pain at the chosen low intensity. *CS:* consisted of a 12 min baseline and 12 min test period, in which the CS tone (3kHz, 70dB; pre-conditioned with footshocks) was presented 10 times for 20 s each time. The inter-CS interval was either 60 or 90 s in a pseudorandomized order.

##### Testing in the muscimol experiment

Testing in the muscimol experiment was composed of two conditions: HighShockObs and CS. *HighShockObs*: three days after pre-exposure the HighShockObs test was performed. Fifteen minutes prior to the shock observation test, observer animals were lightly restrained, the stylet was removed and an injection cannula (62201; RWD Life Science Co., Ltd) extending 0.8 mm below the guide cannula was inserted. Muscimol (0.1 μg/μl) or saline (0.9%) was microinjected using a 10 μL syringe (Hamilton), which was attached to the injection cannula by PE 20 tubing (BTPE-20; Instech Laboratories, Inc.). A volume of 0.5 μL per side was injected using a syringe pump (70-3007D; Harvard Apparatus Co.) over a 60 s period, and the injection cannula remained untouched for an additional 60 s to allow for absorption into the brain region and to minimize injectate along the track of the cannula. The protective cap was secured to the observer animal after the infusion and the animal was returned to the home cage. Six (2 from saline and 4 from muscimol group) were excluded due to damaged or clogged cannulas. Shock observation test then started with a 12-minute baseline, followed by the observer witnessing the demonstrator experience 5 footshocks (1sec, 1.5mA each, pseudorandom intershock interval 120 or 180 s). *CS*: One week later, the observers underwent the conditioned stimulus recall test. Fifteen minutes prior to the test, microinjection of muscimol or saline were performed with the same protocol as prior to the shock observation test. Observers were then put into a skinner box in a context that was different from the shock observation test (i.e., different smell, illumination, and floor texture). After 12 min baseline, the CS tone were played for 5 times (20 s each, 120 or 180 s pseudorandom interval). All test sessions were videotaped using a Basler GigE camera (acA1300-60 gm) controlled by MediaRecorder 2 (Noldus, the Netherlands).

##### Histology

For the electrophysiology experiment, after completion of the experiment, animals were deeply anesthetized and an electrical lesion was performed to mark the positions of the electrodes (2μA, 10 s, across the top and bottom most contact of each leg). For both experiments animals were intracardially perfused with phosphate buffered saline (PBS, 7.4pH) followed by 4% paraformaldehyde, brains were removed, cut with a cryostat (50μm coronal sections), and Nissl stained for verification of shafts track and electrode positioning for electrophysiology experiment and cannula position for muscimol experiment. For the muscimol experiment four dyads (2 from saline group and 2 from muscimol group) were excluded from data analyses after histology examination suggesting damage of corpus callosum due to injection.

### Quantification and Statistical Analysis

#### Sample size calculation

For the electrophysiology experiment, based on results from previous studies in action mirror neurons [48] together with preliminary data, it was estimated that approximately 30% of cells would respond to pain experience and 10% of them would be emotional mirror neurons (i.e., 3 emotional mirror cells per 100 cells). We aimed to record from about 30 mirror cells to document the existence of these cells and characterize their functionality. Pilot studies with silicon probes showed that on average 2 emotional mirror cells were obtained per animal, thus to obtain ca. 30 mirror cells, a total of 15 observers was required. In addition, 2 more animals were added to this estimate to take into account a 10% animal loss due to either surgical complications or problems with the probes. This resulted in a total of 17 observers, each paired with a different demonstrator, resulting in a total of 34 animals.

For the muscimol experiment, based on a similar experiment on empathy in mice [16] using percent freezing of observer animals with lidocaine micro injection in ACC before test compared to control animals that had saline injection, the μ1- μ0 = 33 and the standard deviation = 27 (corresponds to a cohen’s d effect size = 1.22). Although this study used mice, not rats, we used this effect size as an estimate of the effect size we might find in rats. With an effect size of 1.22, alpha = 0.05, and power = 0.8 (t test, one sided), and 15% expected loses (for surgical complications or miss target), power analysis resulted in 15 pairs of animals per group. Therefore 15 pairs ^∗^2 groups = 30 pairs of animals were required. Each pair includes 1 observer and 1 demonstrator animal so in total 30 pairs ^∗^2 animals/pair (observer +demonstrator) = 60 animals were used.

#### Data acquisition

The electrophysiology signals (continuous and single units) were unit gain amplified using a head stage pre-amplifier (HS-36, Neuralynx), relayed to an input board differential input amplifiers with a gain of 15 and acquired with a sampling frequency of 32kHz. For single units, the signal was bandpass filtered (0.6-6 kHz), timestamped and recorded for 1 ms every time the signal passed a manually set threshold.

#### Data analysis

##### Behavioral data

The onset and duration of the observer and demonstrator behavior during the tests were manually scored offline in a continuous manner using the open source Behavioral Observation Research Interactive Software (BORIS, [49]) and analyzed using MATLAB (MathWorks Inc., USA). For the electrophysiology experiment, the shock observation test, freezing, rearing, head location, jumping and head orientation of the observer and demonstrator were scored. The observer’s head orientation relative to the line connecting the heads of the two animals was used to quantify attention (Figure 4F), while the distance between the head of the observer and the demonstrator’s cabinet was used to measure proximity. Soundtracks of the session were also analyzed using MATLAB to extract power per frequency (0-80kHz) relative to the onsets of each condition (Figure S2). Specifically, the time-frequency decomposition was averaged over all trials of a given condition, and conditions were then compared by performing a t test separately at each time and frequency by including one value per animal akin to the random effect mass-univariate analyses typical of neuroimaging data. For the laser experience test, the reaction of the observer to the stimuli was scored offline to confirm successful laser targeting. Trials with no behavioral reaction to HighLaser were excluded from further analyses. For 6 animals, the video recorded were missing frames, leading to a misalignment of the video with the event triggers, making further behavioral analysis difficult. For the remaining 11 animals (7 of which had also the LowLaser condition), we performed additional behavioral scorings. First, we rated freezing manually as described above, and calculated the proportion of time spent freezing for each condition in the interval from the beginning of that condition (e.g., HighLaser) until the beginning of the next condition (e.g., CtrlLaser). Additionally, we used Noldus et al. [50] software to track the rat’s motion continuously. Finally, author YH rated each trial as showing one of 4 levels of response intensity: 0 = no response, 1 = mild response (e.g., head movements), 2 = medium response (paw movement/licking), 3 = intense response (vigorous paw and body movement). Results are shown in Figure S2. For the CS recall test we scored freezing, grooming and laying down (without freezing) using BORIS, and the proportion of time spent in each behavior is shown for the baseline, the 20 s of CS playback and the ISI between two 20 s CS. This was done separately for the first and last 5 trials to explore habituation. The outcome of these analyses can be found in Figures 4 and S2 and are discussed in the Results section The Demonstrators Squeak and Jump while the ACC Responds Maximally.

For the muscimol experiment freezing time was calculated as the sum of all freezing moments in a certain epoch and freezing percentage was calculated as the total freezing time divided by the total time of the epoch. Baseline period was defined as the first 720 s of the test and the test period was defined as the 720 s after baseline, starting with the first shock or CS playback.

##### Multiunit activity

Multiunit activity analyses were performed using the FieldTrip toolbox [51] and custom-made MATLAB scripts. Data from the 32 contacts were first visually explored to identify artifacts. This was done (a) taking the raw signal from each of the 30 high impedance contacts relative to the low impedance reference channel, high-pass filtering it at 1000 Hz, rectifying and low-pass filtering at 200 Hz to approximate MUA. Trials in which extreme MUA activity (z > 8 or z < −5) occurred across many or all channels were removed. This lead to the rejection of 7 trials in total. Inspection of the video recordings identified 5 more trials that had to be rejected because the Faraday cage had to be opened for experimental reasons. We then performed a pairwise re-referencing of the cleaned data on each electrode shaft offline. Each of the 5 shafts of the electrode had 6 contacts, and re-referencing was done by subtracting raw data from the vertically adjacent contact of each leg resulting in 5 channels (each the difference of two contacts) per shaft, or 25 channels per animal. The data was then high-pass filtered at 1000 Hz, rectified and low-pass filtered at 200 Hz as recommended in [28]. Trial data was extracted from 2 s before to 3 s after the onset of the stimuli.

For statistical analyses, we used the surface under the MUA. Specifically, we computed the area under the MUA during the baseline epoch (−1.2 s to 0.2 s relative to stimulus onset) and during an epoch of same duration (1 s) during stimulus presentation when we expect the response to occur. For Shock and CS trials, we expect low latency responses and therefore used the epoch 0 s to 1 s post stimulus onset. For Laser, three reasons lead us to expect longer latencies: (a) the laser pulse had a 200ms duration and the thermal energy does not reach its maximum (and hence painful level) before 200ms, (b) the burning pain most associated with affective reactions depend on unmyelinated fibers with slow conduction times and (c) laser evoked spiking in the ACC has been reported to reflect laser intensity reliably from ∼300ms after stimulus onset [30, 52]. Accordingly, for that condition we shifted the experimental epoch to 0.3 s to 1.3 s post laser onset. The same duration interval was used in all conditions (1 s) to avoid biasing analyses for a particular condition. t tests were then used to compare responses against baseline or across conditions because the surface under the MUA in 1 s intervals were continuous and approximately normally distributed (i.e., less than 5% of channels failed a Kolmogorov-Smirnov test at p < 0.05). Paired t test were used when comparing an experimental period against its directly preceding baseline, and two sample t test were used when comparing the experimental period across conditions. The significance threshold was set at 0.01. One-tailed tests were used because we focus on stimulus triggered activations (i.e., increases in MUA). Post hoc, we also explored the presence of stimulus driven reductions of MUA, but found these to be very rare, and we therefore do not further explore them.

We first identified channels that were responsive by requiring that HighLaser > baseline OR HighShockObs > baseline OR CS > Baseline. We then explored the selectivity of those channels that were ‘responsive’ by comparing conditions against their control condition (i.e., HighLaser > CtrlLaser; HighShockObs > CtrlShockObs). For the CS playback we were more lenient in order to detect all cells that respond to any salient sound, and thus compared CS against baseline (CS > baseline). Using the CtrlLaser and CtrlShockObs as reference further ensures that responses cannot be due to the sound produced when triggering the Laser or delivering the Shocks, as these are also present in the Ctrl conditions. The outcome of the analysis on the MUAs can be found in the 1^st^-3^rd^ paragraphs of the Results section and in the legend of Figures 2 and S1.

To look for habituation at the population level, we quantified the MUA for each trial and contact as the area under the curve (AUC) in the response interval. We then averaged this AUC per channel for the first and last 5 trials of each condition. We then entered this data into an ANOVA with 3 conditions (HighShockObs, HighLaser, CS) and 2 epochs (First 5 trials, Last 5 trials), and found a significant interaction, which was followed-up by pairwise t tests for each condition. Results are shown in Figure S1.

##### Single unit data

To characterize the response of units that respond to witnessing a shock to another animal, the data from the ShockObs session were clustered using spikesort 3D (Neuralynx) and KlustaKwik [53] then manually examined to identify the channels in which there were single units present. Channels without single unit activity were excluded from further processing. To ensure that the same single unit was present across all sessions (i.e., ShockObs, Laser & CS) data from each contact from the three sessions was merged into a single file and processed and analyzed as if it was a unique session. This merged data was then automatically clustered and manually cleaned using SpikeSort 3D and KlustaKwik. To further ensure that the same cell was present across sessions, spike waveforms and firing rate during baseline period of the Laser and CS recall sessions were compared to the shock session and a cleanup procedure was conducted as follows: 1) spikes with a waveform-correlation lower than 0.85 with the average shock waveform were removed, 2) spikes with a peak amplitude beyond ± 15% of the peak amplitude of the average shock waveform were removed and 3) sessions with a firing rate ratio between the Shock baseline period and the other sessions’ baseline period higher than 8 were removed (after steps 1 and 2 had been applied). In addition, sessions with a spike firing rate lower than 0.06Hz were not included in the final analysis.

Statistical analyses for the electrophysiology experiment were performed on spike counts from epochs defined as for the MUA analysis: baseline epoch were always from −1.2 to −0.2 s relative to stimulus onset, and experimental epochs were from 0 to 1 s for Shock and CS, and 0.3 to 1.3 s for Laser. Spike counts were compared against their baseline using one-tailed Wilcoxon signed-rank test, threshold set at p < 0.05. This non-parametric test was used instead of a t test at p < 0.01 because spike counts are discrete numbers that are sometimes ill distributed. However analyzing the same data with t tests at p < 0.01 lead to conceptually similar results. Spike counts were compared across conditions using the Wilcoxon rank sum test at p < 0.05. Clusters were classified as responsive in a given session, if HighShockObs > Shock Baseline OR HighLaser > Laser Baseline OR CS > CS Baseline. For shock and laser sessions, clusters were classified as specific in their response in a given condition if HighShockObs > CtrlShockObs or HighLaser > CtrlLaser. As for the MUA, CS responses were simply assessed compared to baseline to be sensitive in detecting any response to a salient event. The results of the single unit analysis can be found in paragraphs 5 and 6 of the Results section and in Figure 3.

To illustrate the average response of pain-selective mirror neurons, we selected the 23 neurons that had the following properties: HighShockObs > CtrlShockObs & HighLaser > CtrlLaser and HighLaser > CS (all at p < 0.05). We then calculated the spike density function (sdf) for each of these neurons separately (using an exponential decay function of 200ms and a Gaussian of 50ms and the function msdf from MLIB toolbox for MATLAB). To normalize the firing rate, we determine the peak of the sdf across all conditions within the experimental window (0-1 s for CS and ShockObs and 0.3-1.3 s for Laser). For each condition, we then removed baseline activity (averaged from −2.2 s to −1.2). Then we divided the de-baselined sdf of each condition by the same value: the maximum sdf across all conditions. This ensures that for each cell, a value of 1 represents the maximum firing rate across all conditions. Then we averaged all 23 neurons, and show the average normalized sdf together with the standard error of the mean in Figure 3.

For the muscimol experiment, for comparison between periods (baseline versus shock or CS) and conditions (muscimol versus control), repeated-measures ANOVAs (IBMSPSS statistics, USA) were performed with baseline and test period as within subject factors and the conditions as between-subject factors. The outcome of this analysis can be found in the last paragraph of the Results section and in the legend of Figure 5.

##### Spike triggered spectrogram

To further characterize the features triggering the activity of interesting neurons (Figure 3A6) in the Shock session we performed spike triggered analyses of the sound recording. For this analysis, we did not consider the first 12 min of baseline but focused on the continuous period starting shortly before the first shock and ending after the last shock. In that period, instantaneous firing rate of each neuron was calculated as the inverse of the inter-spike interval. Moments of unusual firing were then identified as moments with instantaneous firing rate in the top 5% of all instantaneous firing rates. For each of these moments, we then extracted the 5 s prior and after the surprising spike rate and averaged this spectrogram across all surprising spike rates. Finally, to identify *changes* of power associated with the spikes, we subtract for each moment at a given frequency the average power in that frequency across the whole experimental period. The results for the spike triggered spectrogram can be found in Figure 3.

##### Cross-Modal decoding

We used a penalized maximum likelihood generalized linear model to analyzed the spike counts of the 69 neurons for which we have 10 trials of the High, Low and Ctrl conditions for ShockObs and Laser. We used the package glmnet version 2.0 as implemented in R (https://cran.r-project.org/web/packages/glmnet/index.html). We coded stimulus amplitude as 0 = Ctrl, 1 = Low, 2 = High, and used the spike count in the experimental window (0-1 s post shock for ShockObs and 0.3-1.3 s for Laser) for each of the 10 trials at each intensity as the input for the regression and fit the glmnet for optimal leave one out cross-validation in the training cmodality (e.g., train on ShockObs). We then estimate the performance within that modality as the correlation between leave-one-out predicted and actual stimulus intensity. Without refitting, we then use the optimal model from the training modality to predict stimulus intensity based on spike counts from the other modality (e.g., test on Laser), and again calculate the correlation between predicted and actual intensity. Results are presented in the section Common Coding in the ACC.

### Data and Software Availability

Most data and analysis scripts can be found on Mendeley Data at https://doi.org/10.17632/rkt957v4n4.1. Raw data can be obtained from the Lead Contact.

## Supplemental Information

Document S1. Figures S1–S3

Document S2. Article plus Supplemental Information

## References

[1] de Waal F.B.M., Preston S.D. (2017). Mammalian empathy: behavioural manifestations and neural basis. Nat. Rev. Neurosci..

[2] Lamm C., Decety J., Singer T. (2011). Meta-analytic evidence for common and distinct neural networks associated with directly experienced pain and empathy for pain. Neuroimage.

[3] Singer T., Seymour B., O’Doherty J., Kaube H., Dolan R.J., Frith C.D. (2004). Empathy for pain involves the affective but not sensory components of pain. Science.

[4] Meffert H., Gazzola V., den Boer J.A., Bartels A.A.J., Keysers C. (2013). Reduced spontaneous but relatively normal deliberate vicarious representations in psychopathy. Brain.

[5] Rütgen M., Seidel E.-M., Silani G., Riečanský I., Hummer A., Windischberger C., Petrovic P., Lamm C. (2015). Placebo analgesia and its opioidergic regulation suggest that empathy for pain is grounded in self pain. Proc. Natl. Acad. Sci. USA.

[6] Mischkowski D., Crocker J., Way B.M. (2016). From painkiller to empathy killer: acetaminophen (paracetamol) reduces empathy for pain. Soc. Cogn. Affect. Neurosci..

[7] Gallese V., Keysers C., Rizzolatti G. (2004). A unifying view of the basis of social cognition. Trends Cogn. Sci..

[8] Hutchison W.D., Davis K.D., Lozano A.M., Tasker R.R., Dostrovsky J.O. (1999). Pain-related neurons in the human cingulate cortex. Nat. Neurosci..

[9] Sakaguchi T., Iwasaki S., Okada M., Okamoto K., Ikegaya Y. (2018). Ethanol facilitates socially evoked memory recall in mice by recruiting pain-sensitive anterior cingulate cortical neurons. Nat. Commun..

[10] Zaki J., Wager T.D., Singer T., Keysers C., Gazzola V. (2016). The anatomy of suffering: understanding the relationship between nociceptive and empathic pain. Trends Cogn. Sci..

[11] Wager T.D., Atlas L.Y., Botvinick M.M., Chang L.J., Coghill R.C., Davis K.D., Iannetti G.D., Poldrack R.A., Shackman A.J., Yarkoni T. (2016). Pain in the ACC?. Proc. Natl. Acad. Sci. USA.

[12] Krishnan A., Woo C.-W., Chang L.J., Ruzic L., Gu X., López-Solà M., Jackson P.L., Pujol J., Fan J., Wager T.D. (2016). Somatic and vicarious pain are represented by dissociable multivariate brain patterns. eLife.

[13] Corradi-Dell’Acqua C., Tusche A., Vuilleumier P., Singer T. (2016). Cross-modal representations of first-hand and vicarious pain, disgust and fairness in insular and cingulate cortex. Nat. Commun..

[14] Atsak P., Orre M., Bakker P., Cerliani L., Roozendaal B., Gazzola V., Moita M., Keysers C. (2011). Experience modulates vicarious freezing in rats: a model for empathy. PLoS ONE.

[15] Carrillo M., Migliorati F., Bruls R., Han Y., Heinemans M., Pruis I., Gazzola V., Keysers C. (2015). Repeated witnessing of conspecifics in pain: effects on emotional contagion. PLoS ONE.

[16] Jeon D., Kim S., Chetana M., Jo D., Ruley H.E., Lin S.-Y., Rabah D., Kinet J.-P., Shin H.-S. (2010). Observational fear learning involves affective pain system and Cav1.2 Ca^2+^ channels in ACC. Nat. Neurosci..

[17] Gonzalez-Liencres C., Juckel G., Tas C., Friebe A., Brüne M. (2014). Emotional contagion in mice: the role of familiarity. Behav. Brain Res..

[18] Keum S., Shin H.-S. (2016). Rodent models for studying empathy. Neurobiol. Learn. Mem..

[19] Kim S., Mátyás F., Lee S., Acsády L., Shin H.-S. (2012). Lateralization of observational fear learning at the cortical but not thalamic level in mice. Proc. Natl. Acad. Sci. USA.

[20] Sanders J., Mayford M., Jeste D. (2013). Empathic fear responses in mice are triggered by recognition of a shared experience. PLoS ONE.

[21] Gallese V., Fadiga L., Fogassi L., Rizzolatti G. (1996). Action recognition in the premotor cortex. Brain.

[22] Thioux M., Gazzola V., Keysers C. (2008). Action understanding: how, what and why. Curr. Biol..

[23] Vogt B., Paxinos G. (2015). Cingulate cortex and pain architecture. The Rat Nervous System.

[24] Allsop S.A., Wichmann R., Mills F., Burgos-Robles A., Chang C.-J., Felix-Ortiz A.C., Vienne A., Beyeler A., Izadmehr E.M., Glober G. (2018). Corticoamygdala transfer of socially derived information gates observational learning. Cell.

[25] Pereira A.G., Cruz A., Lima S.Q., Moita M.A. (2012). Silence resulting from the cessation of movement signals danger. Curr. Biol..

[26] Cruccu G., Sommer C., Anand P., Attal N., Baron R., Garcia-Larrea L., Haanpaa M., Jensen T.S., Serra J., Treede R.-D. (2010). EFNS guidelines on neuropathic pain assessment: revised 2009. Eur. J. Neurol..

[27] Bromm B., Treede R.D. (1991). Laser-evoked cerebral potentials in the assessment of cutaneous pain sensitivity in normal subjects and patients. Rev. Neurol. (Paris).

[28] Supèr H., Roelfsema P.R. (2005). Chronic multiunit recordings in behaving animals: advantages and limitations. Prog. Brain Res..

[29] Stark E., Abeles M. (2007). Predicting movement from multiunit activity. J. Neurosci..

[30] Zhang Y., Wang N., Wang J.-Y., Chang J.-Y., Woodward D.J., Luo F. (2011). Ensemble encoding of nociceptive stimulus intensity in the rat medial and lateral pain systems. Mol. Pain.

[31] Fan R.-J., Kung J.-C., Olausson B.A., Shyu B.-C. (2009). Nocifensive behaviors components evoked by brief laser pulses are mediated by C fibers. Physiol. Behav..

[32] LeDoux J.E. (2014). Coming to terms with fear. Proc. Natl. Acad. Sci. USA.

[33] Paxinos G., Watson C. (2013).

[34] Martin J.H. (1991). Autoradiographic estimation of the extent of reversible inactivation produced by microinjection of lidocaine and muscimol in the rat. Neurosci. Lett..

[35] Han Y., Bruls R., Thomas R.M., Pentaraki V., Jelinek N., Heinemans M., Bassez I., Verschooren S., Pruis I., van Lierde T. (2018). Cingulate dependent social risk assessment in rats. bioRxiv.

[36] Jourdan D., Ardid D., Chapuy E., Eschalier A., Le Bars D. (1995). Audible and ultrasonic vocalization elicited by single electrical nociceptive stimuli to the tail in the rat. Pain.

[37] Tovote P., Fadok J.P., Lüthi A. (2015). Neuronal circuits for fear and anxiety. Nat. Rev. Neurosci..

[38] Giustino T.F., Maren S. (2015). The role of the medial prefrontal cortex in the conditioning and extinction of fear. Front. Behav. Neurosci..

[39] Herry C., Ciocchi S., Senn V., Demmou L., Müller C., Lüthi A. (2008). Switching on and off fear by distinct neuronal circuits. Nature.

[40] Keysers C., Gazzola V. (2014). Hebbian learning and predictive mirror neurons for actions, sensations and emotions. Philos. Trans. R. Soc. Lond. B Biol. Sci..

[41] Keysers C., Perrett D.I. (2004). Demystifying social cognition: a Hebbian perspective. Trends Cogn. Sci..

[42] Church R.M. (1959). Emotional reactions of rats to the pain of others. J. Comp. Physiol. Psychol..

[43] Greene J.T. (1969). Altruistic behavior in the albino rat. Psychon. Sci..

[44] Ben-Ami Bartal I., Decety J., Mason P. (2011). Empathy and pro-social behavior in rats. Science.

[45] Kohler E., Keysers C., Umiltà M.A., Fogassi L., Gallese V., Rizzolatti G. (2002). Hearing sounds, understanding actions: action representation in mirror neurons. Science.

[46] Keysers C., Gazzola V. (2017). A plea for cross-species social neuroscience. Curr. Top. Behav. Neurosci..

[47] Keysers C., Gazzola V. (2009). Expanding the mirror: vicarious activity for actions, emotions, and sensations. Curr. Opin. Neurobiol..

[48] Keysers C., Wicker B., Gazzola V., Anton J.-L., Fogassi L., Gallese V. (2004). A touching sight: SII/PV activation during the observation and experience of touch. Neuron.

[49] Friard O., Gamba M. (2016). BORIS: a free, versatile open-source event-logging software for video/audio coding and live observations. Methods Ecol. Evol..

[50] Noldus L.P., Spink A.J., Tegelenbosch R.A. (2001). EthoVision: a versatile video tracking system for automation of behavioral experiments. Behav. Res. Methods Instrum. Comput..

[51] Oostenveld R., Fries P., Maris E., Schoffelen J.M. (2011). FieldTrip: open source software for advanced analysis of MEG, EEG, and invasive electrophysiological data. Comput. Intell. Neurosci..

[52] Zhang Q., Xiao Z., Huang C., Hu S., Kulkarni P., Martinez E., Tong A.P., Garg A., Zhou H., Chen Z., Wang J. (2018). Local field potential decoding of the onset and intensity of acute pain in rats. Sci. Rep..

[53] Kadir S.N., Goodman D.F.M., Harris K.D. (2014). High-Dimensional Cluster Analysis with the Masked EM Algorithm. Neural Comput..

