# Supplementary material for: Emotional Mirror Neurons in the Rat’s Anterior Cingulate Cortex
Source: Curr Biol. 2019 Apr 22;29(8):1301–1312.e6. doi: 10.1016/j.cub.2019.03.024 (PMC6488290; doi:10.1016/j.cub.2019.03.024)
Supplement: Document S1. Figures S1–S3 [file mmc1.pdf]

Current Biology, Volume *29*

Supplemental Information

Emotional Mirror Neurons

in the Rat's Anterior Cingulate Cortex

Maria Carrillo, Yinging Han, Filippo Migliorati, Ming Liu, Valeria Gazzola, and Christian Keysers

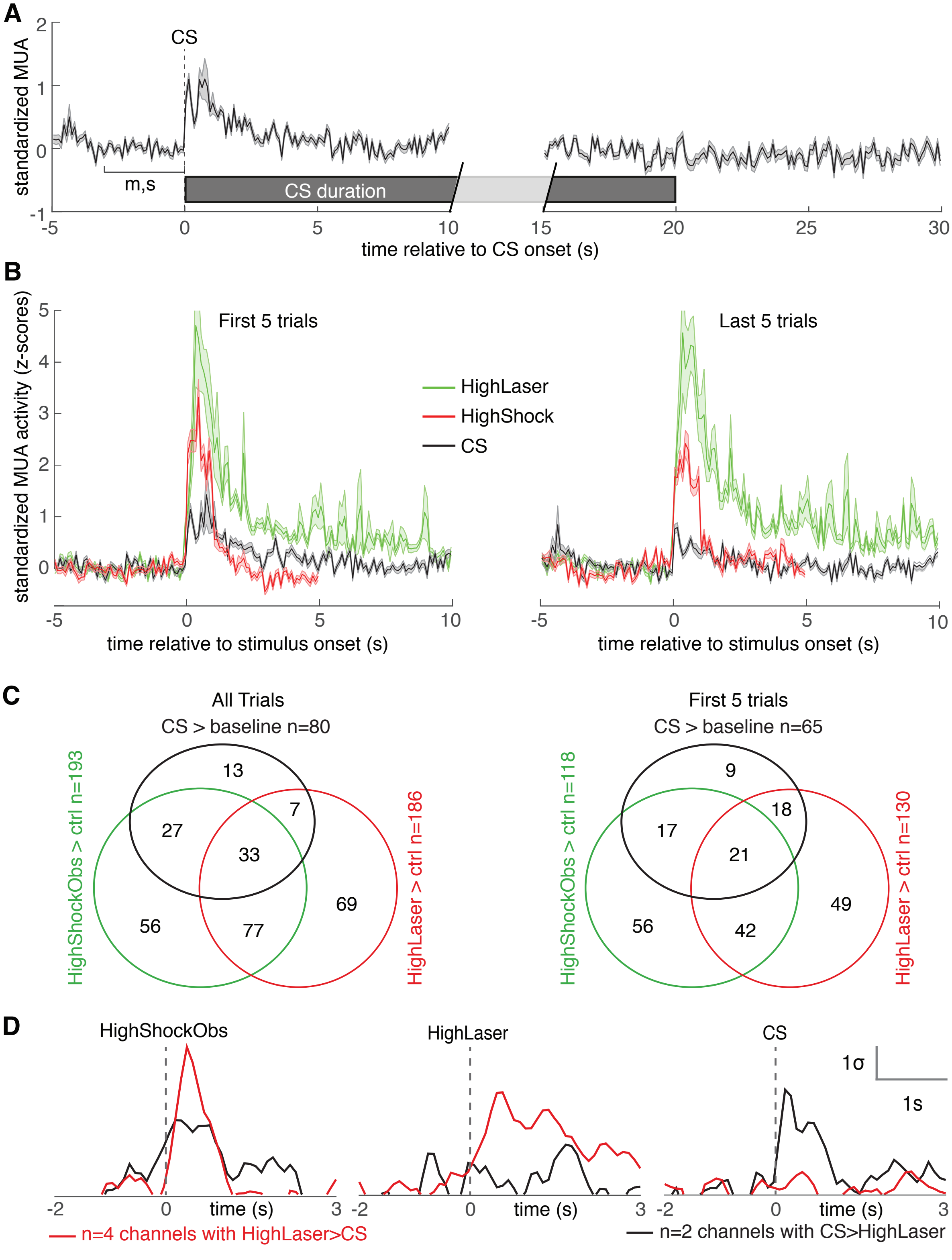

Figure S1 Multiunit Activity and its habituation. Related to Figure 2. (A) Mean (+/-sem) z-transformed MUA aligned on the beginning of the 20s CS (t=-5 to 10s) and its end (15-30s). Both epochs were z-transformed relative to mean and standard deviation of the same pre-stimulus baseline shown with a square bracket. **(B)** Population MUA as in Figure 2G,H separately for the first 5 and last 5 trials to examine habituation. See main text for statistical analysis. **(C)** Left: Classification of MUA channels as in Figure 2F, Right: same but for the first 5 trials only. **(D)** Average MUA response of two small populations of MUA channels responding to HighShockObs that were recorded simultaneously in one animal (#31) and had opposite preferences for HighLaser vs. CS. The red line represents the average of 4 channels that responded to HighLaser>CtrlLaser ($p<0.01$ for each channel) but not CS>Baseline ($p>0.2$ for each channel). The blue line represents the average of 2 channels that responded to CS>Baseline (all $p<0.01$) but not HighLaser>CtrlLaser (all $p>0.2$). All 6 channels showed significant responses to ShockObs (HighShockObs>CtrlShockObs, all $p<0.01$). The time courses illustrate how within the same animal, simultaneously recorded channels can share a sensitivity to the signals of another rat but show opposite selectivity during self experience. Given that they were recorded simultaneously, this cross-over cannot be explained by the animal failing to notice one condition altogether.

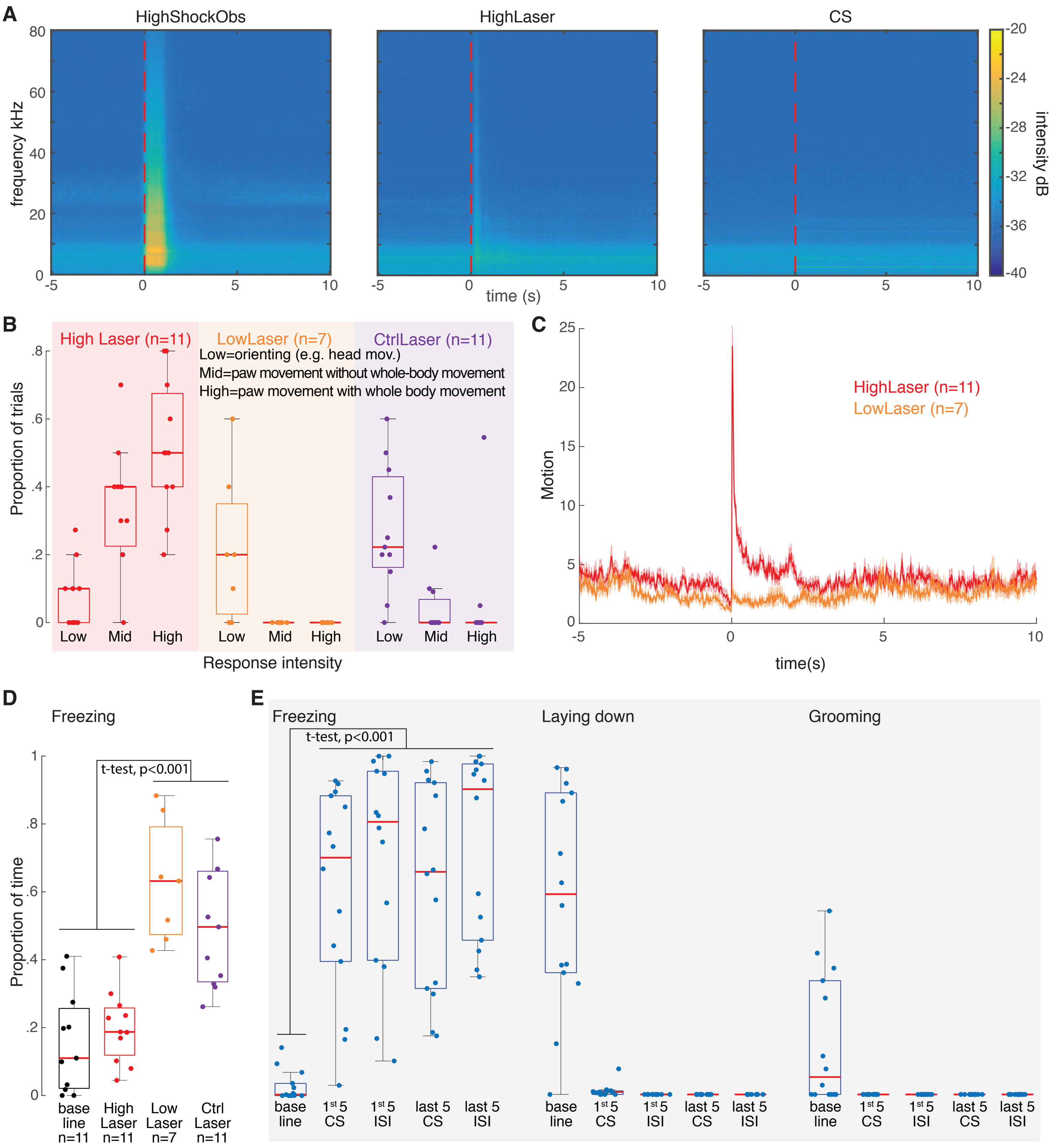

Figure S2: Behavioral Analysis. Related to Figure 4. (A) The three conditions had different spectrograms. HighShockObs was characterized by squeaking in the audible range (left), HighLaser by a subtle sound resulting from the rat's bodily movements (e.g. paw retraction), CS by the 3kHz CS and its harmonics. **(B)** The immediate behavioral reaction to Laser application was manually rated as absent, low, mid or high intensity and the proportion of trials with that intensity plotted for each animal (dot) as a function of Laser condition with quartiles as wiskerplot. Mid and High intensity reactions were more frequent in the HighLaser condition (Wilcoxon signed rank vs. Ctrl and Low, all p<0.01), but there was no significant difference to Low vs. CtrlLaser (Wilcoxon signed rank, all p>0.8). **(C)** Motion in the recorded videos in 100ms intervals for the High and Lowlaser condition (mean±sem). **(D)** Proportion freezing was higher following Low and CtrlLaser than baseline and HighLaser. The vigorous HighLaser reactions (B,C) led to freezing close to baseline levels. Note: due to temporal misalignment between movies and event-marker, all behavioral Laser analyses (B-D) are restricted to a subset of animals as indicated, and non-parametric stats were used because of normality violation. **(E)** Three behaviors were analyzed separately during baseline, CS playback and the interstimulus interval (ISI) between CS playbacks, separately for the 1st and last 5 trials to examine habituation. Freezing was elevated relative to baseline (paired t-test, all p<0.001). A 2 way repeated measurement ANOVA with CS vs ISI and first vs. last 5 trials revealed a main effect of CS vs ISI (F(1,13)=14.4, p<0.002) but no habituation (first vs last, F(1,13)=0.5, p=0.48) or interaction (F(1,13)=0.5, p=0.48). CS playback caused freezing to replacing grooming and laying down. All CS analyses were performed on n=14 aninals for which the movies were temporally well aligned with the event markers, and parametric stats were used because normality was not violated.

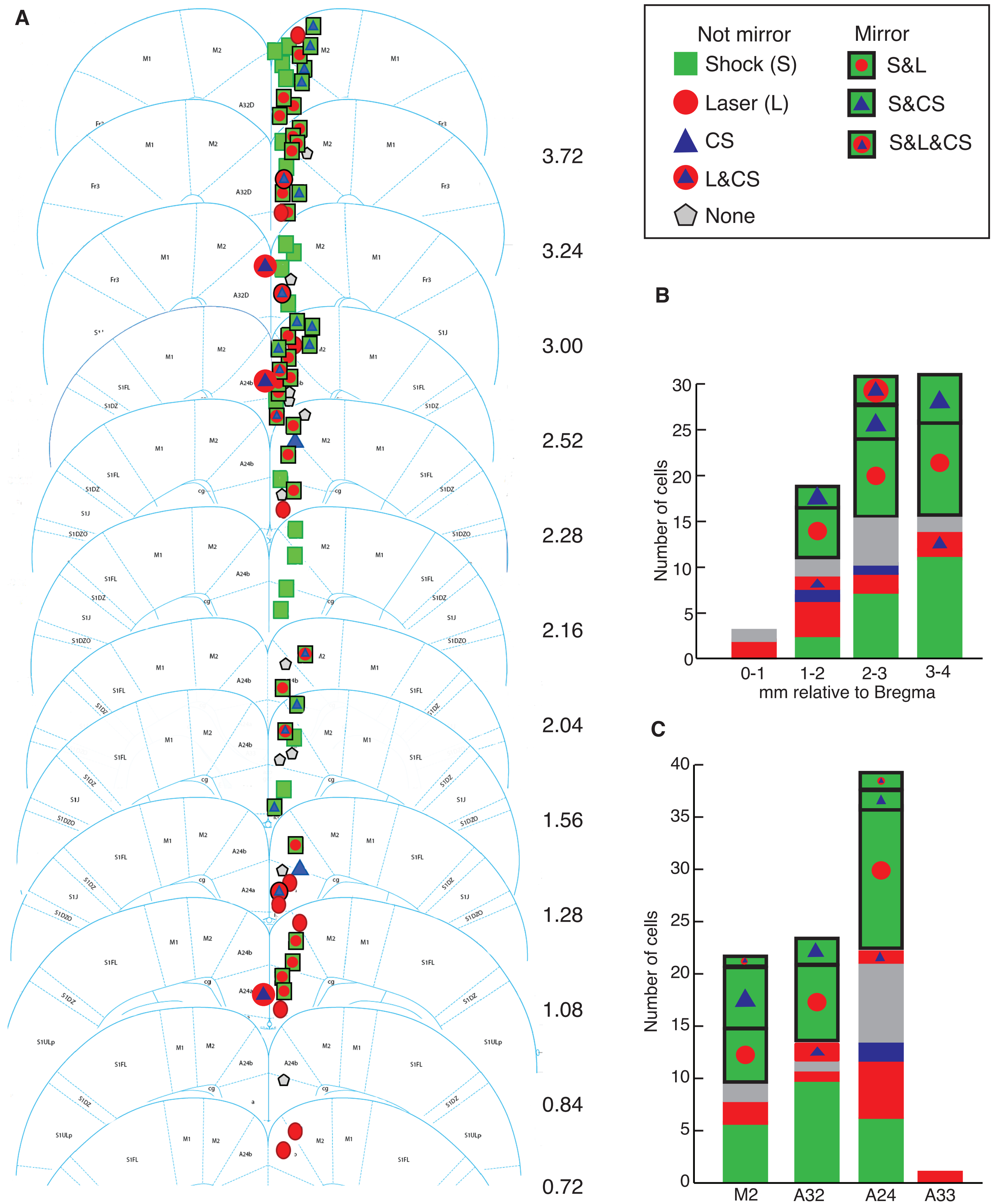

Figure S3: Histological reconstruction of single cells. Related to Figure 3. (A) On coronal slices with Bregma AP in mm adapted from Ref. [S1]. **(B-C)** Cell types as a function of AP coordinates (B) or brain region (C). No significant difference in the proportion of mirror cells (S&L vs S&CS vs S&L&CS) were found across AP ($X^2(9)= 8.27$, $p=0.5$) or brain region ($X^2(9)=8.73$, $p=0.46$).

Supplemental References

S1. Paxinos, G., and Charles Watson (2014). The Rat Brain in Stereotaxic Coordinates 7th Edition 7th ed. (Amsterdam, The Netherlands: Elsevier).
